# Dental Deposits Are Differentially Associated With Periodontal Conditions and the Number of Teeth in Japanese Community‐Dwelling Individuals: The Nagasaki Islands Study

**DOI:** 10.1002/cre2.70101

**Published:** 2025-03-03

**Authors:** Masayuki Oohira, Masayasu Kitamura, Kanako Higuchi, Mark Luigi Fabian Capati, Mami Tamai, Saki Ichinose, Yasunori Yamashita, Yukio Ozaki, Eijiro Sakamoto, Yumiko Kawashita, Sakiko Soutome, Takahiro Maeda, Atsushi Kawakami, Toshiyuki Saito, Atsutoshi Yoshimura

**Affiliations:** ^1^ Department of Periodontology and Endodontology Nagasaki University Graduate School of Biomedical Sciences Nagasaki Japan; ^2^ Department of Oral Health Nagasaki University Graduate School of Biomedical Sciences Nagasaki Japan; ^3^ Department of Immunology and Rheumatology Nagasaki University Graduate School of Biomedical Sciences Nagasaki Japan; ^4^ Leading Medical Research Core Unit Nagasaki University Graduate School of Biomedical Sciences Nagasaki Japan; ^5^ Department of General Medicine Nagasaki University Graduate School of Biomedical Sciences Nagasaki Japan

**Keywords:** dental deposits, epidemiology, periodontal diseases, tooth loss

## Abstract

**Objective:**

This study aimed to determine how dental deposits are associated with periodontal conditions and the number of teeth in Goto Islands' residents.

**Background:**

Previous studies have shown that dental deposits increase the risk of developing periodontal diseases. However, the relationships between dental deposits and the periodontal/dentitional conditions in a super‐aging society remain unclear.

**Materials and Methods:**

A cross‐sectional study involving 671 participants (age: 65.0 ± 12.0 years) was conducted using data from the Nagasaki Islands Study (NaIS). Participants underwent a routine medical examination. Information on oral hygiene and smoking status was collected from a self‐administered questionnaire. Dental examinations were conducted to determine the number of teeth, probing pocket depth (PPD), clinical attachment level (CAL), bleeding on probing (BOP) ratio, calculus index (CI) score, and debris index (DI) score. Saliva samples were collected from the participants to determine the levels of *Porphyromonas gingivalis* and *Aggregatibacter actinomycetemcomitans* by real‐time polymerase chain reaction. Multivariable logistic regression analyses were performed to evaluate the relationships between dental deposits and periodontal/dentitional conditions.

**Results:**

Multivariable logistic regression analyses show that greater DI score was significantly associated with higher BOP ratio (OR = 2.51, 95% CI: 1.75–3.61), greater CAL (OR = 1.51, 95% CI: 1.02–2.23), and fewer teeth (OR = 1.70, 95% CI: 1.04–2.76). Greater CI score was significantly associated with a higher BOP ratio (OR = 2.18, 95% CI: 1.47–3.23), deeper PPD (OR = 2.04, 95% CI: 1.22–3.50), and more teeth (OR = 0.13, 95% CI: 0.08–0.23).

**Conclusions:**

Debris and calculus deposition were associated with more severe periodontal conditions, but calculus deposition was strongly associated with more teeth. The association between calculus deposition and more teeth may be an emerging trend in super‐aging societies, and future longitudinal studies are warranted to elucidate the changing relationship between calculus and number of teeth.

## 1 Introduction

Periodontal disease consists mainly of gingivitis, an inflammatory condition of gingiva, and periodontitis, characterized by the destruction of connective and hard tissue (Pihlstrom et al. 2005). Periodontitis is highly prevalent among older adults and occasionally occurs in young individuals (Catunda et al. 2019). Severe periodontitis causes tooth loss (Michaud et al. 2017), thereby adversely affecting the number of teeth (Borges et al. 2013).

The etiology of periodontitis is multifactorial. The disease results from interactions between microbial pathogens, a susceptible host, and environmental risk factors (Kwon et al. 2021). Among these factors, the bacteria in dental biofilm directly initiate the inflammation and destruction of periodontal tissue. Although more than 700 species of bacteria have been identified in the oral cavity (Aas et al. 2005), only a small number of these bacteria are closely implicated in this disease (Paster et al. 2006). *Porphyromonas gingivalis* is one of the bacteria most closely associated with periodontitis (Yang et al. 2004). The abundance of *P. gingivalis* detected in periodontal pockets correlates positively with the probing pocket depth (PPD) (Kawada et al. 2004). *Aggregatibacter actinomycetemcomitans* is frequently detected in lesions of young patients with periodontitis (Faveri et al. 2009). Putative periodontal pathogens cause dysbiosis in dental biofilms and evoke a persistent host immune response in the periodontium. Host inflammatory response has been revealed as the primary cause of periodontal tissue destruction (Curtis et al. 2020). The host responses are modified by various factors, including systemic disease, age, and gender (Timmerman and van der Weijden 2006). Diabetes mellitus is one of the best‐known systemic diseases associated with periodontal disease caused by impaired insulin secretion, resistance to insulin action, or both (Preshaw and Bissett 2019; Goyal and Jialal 2023). Environmental factors have the capacity to alter both microbial flora and host responses. Environmental factors include smoking, dental calculus, malocclusion, and occlusal trauma (Buduneli 2021). Although dental calculus itself is not responsible for the initiation of periodontitis, its rough surface accelerates plaque retention (Roberts‐Harry and Clerehugh 2000).

Previous epidemiological studies reported that individuals with poor oral hygiene had a higher risk of developing periodontitis than those with good oral hygiene (Lertpimonchai et al. 2017). The Simplified Oral Hygiene Index (OHI‐S) is an indicator of oral hygiene status (Greene and Vermillion 1964). It consists of two indices: the debris index (DI) and the calculus index (CI). DI represents the amount of debris, which is associated with the detection frequencies of periodontopathic bacteria (Umeda et al. 2004). CI represents the amount of calculus, which acts as a reservoir for biologically active and harmful irritants (Cobb and Sottosanti 2021). However, the causes of tooth loss and care for remaining teeth seem to be changing in the current super‐aging society (Kanasi et al. 2016). 29% of Japan's population is ≥ 65 years of age, and aging is even more relevant in remote islands; 42.7% of Goto city's population is ≥ 65 years of age, which may reflect the future state of Japan's super‐aging society. Although it is reported that an increase in dental deposits is positively correlated with the progression of periodontal disease and tooth loss, we hypothesized that this relationship might change in a super‐aging society. Therefore, the purpose of this study was to determine the relationships between dental deposits, such as DI and CI scores, and the periodontal conditions and the number of teeth in residents of the Goto Islands. We conducted a cross‐sectional study that involved an annual medical health checkup in Goto city, western Japan (the Nagasaki Islands Study, NaIS) (Miyata et al. 2023). Under the guidance of Japan's Ministry of Health, Labour and Welfare, Goto city has been promoting medical examinations of community‐dwelling adults for the screening and treatment of non‐communicable diseases. We conducted a dental examination and collected saliva samples from the participants to measure the levels of periodontopathic bacteria, which reflect the bacterial levels in the subgingival plaque (Haririan et al. 2014; Li et al. 2015). The relationship of dental deposits to periodontal and dentitional conditions was statistically analyzed using data obtained from the NaIS (Sekiguchi et al. 2020; Shimizu et al. 2021).

## 2 Materials and Methods

### 2.1 Recruitment of the Study Population

**Figure 1:**
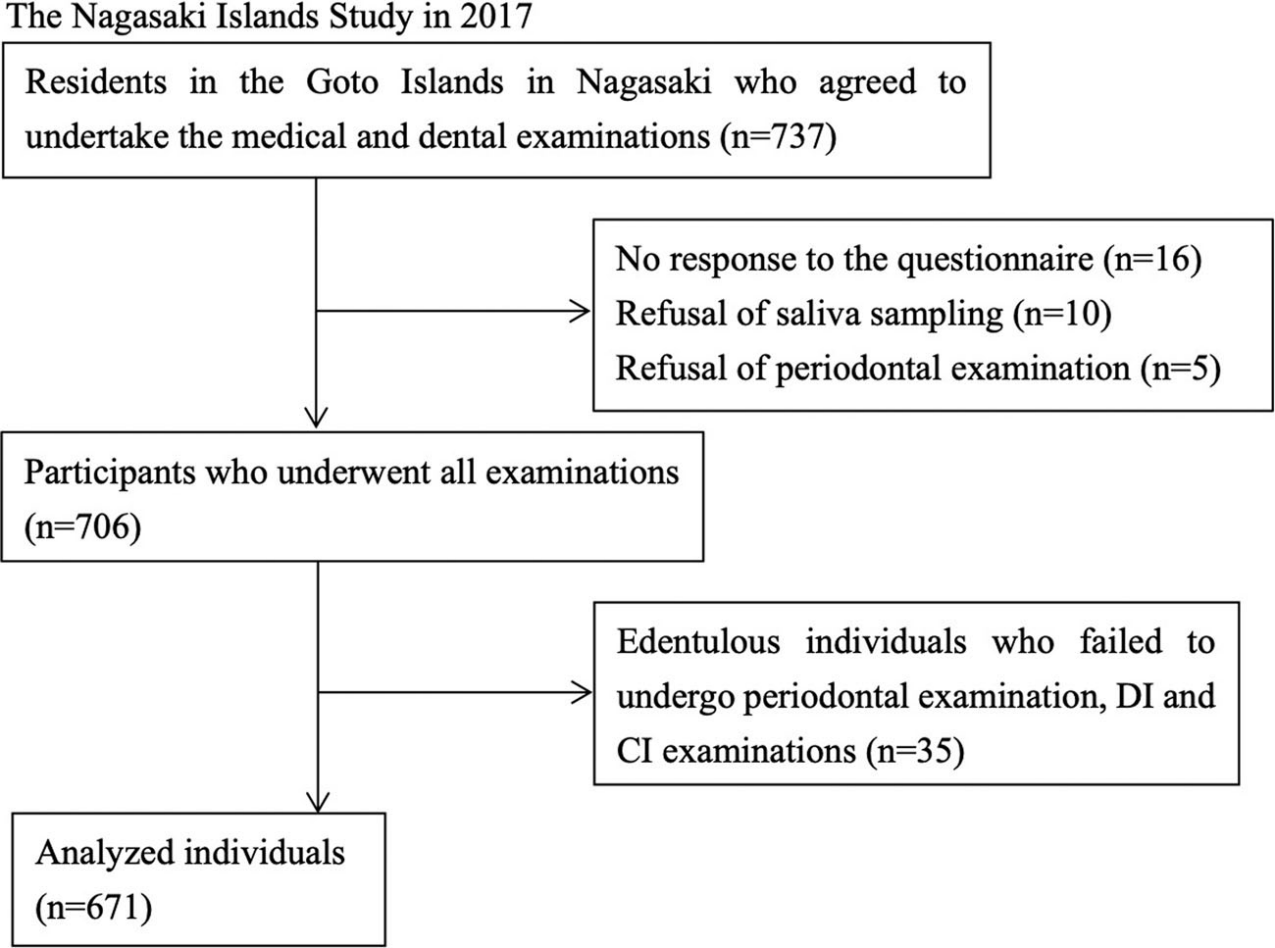
Flow diagram of the study.

In 2017, 737 community‐dwelling adults in the Goto Islands in Nagasaki underwent a medical and dental checkup. We included individuals from this group who agreed to undergo dental examination, including saliva sampling. We excluded those who refused to take the saliva test or periodontal examination or who did not respond to the questionnaire. We also excluded edentulous individuals because periodontal examination and DI and CI examinations could not be performed. (Figure 1). The final study population consisted of 671 participants, the characteristics of whom are shown in Table 1. This study was conducted in accordance with the tenets of the Declaration of Helsinki 2013, and the protocol was approved by the Nagasaki University Ethics Committee (#14051404‐16). All participants received verbal and written information before their participation and provided written informed consent.

**Table 1:** Characteristics of the study population.

Variables	
Participants, *n*	671
Age (years, mean ± SD)	65.0 ± 12.0
Gender (male/female)	236/435
Regular dental checkups (yes/no)	180/491
Frequency of brushing	
< 2 times a day	120
≥ 2 times a day	551
Interdental brushing (yes/no)	368/303
Smoking (yes/no)	54/617
HbA1c (%, mean ± SD)	5.68 ± 0.48
HbA1c ≥ 6.5 (*n* = 41)	6.99 ± 0.05
HbA1c < 6.5 (*n* = 630)	5.60 ± 0.01
Periodontal condition	
BOP (%, mean ± SD)	13.5 ± 18.7
BOP ≥ 10 (*n* = 270)	29.8 ± 0.8
BOP < 10 (*n* = 401)	2.53 ± 0.6
Max PPD (mm, mean ± SD)	4.0 ± 1.8
≥ 7 mm (*n* = 67)	8.0 ± 0.1
5–6 mm (*n* = 129)	5.4 ± 0.1
3–4 mm (*n* = 404)	3.3 ± 0.0
< 3 mm (*n* = 71)	1.9 ± 0.1
Max CAL (mm, mean ± SD)	5.3 ± 2.2
≥ 7 mm (*n* = 153)	8.4 ± 0.1
5–6 mm (*n* = 219)	5.5 ± 0.1
3–4 mm (*n* = 287)	3.6 ± 0.1
< 3 mm (*n* = 12)	1.8 ± 0.3
DI (max) (*n*)	
0	123
1	308
2	182
3	58
CI (max) (*n*)	
0	269
1	271
2	94
3	37
Number of teeth (mean ± SD)	21.8 ± 6.8
< 20 (*n* = 176)	12.1 ± 0.3
≥ 20 (*n* = 495)	25.2 ± 0.2
Bacterial levels (base 10 logarithm)	
*P. gingivalis* (mean ± SD)	2.3 ± 1.9
*A. actinomycetemcomitans* (mean ± SD)	0.4 ± 2.1
Total bacteria (mean ± SD)	5.8 ± 0.7

Abbreviations: BOP, bleeding on probing; CAL, clinical attachment level; CI, calculus index; DI, debris index; HbA1c, hemoglobin A1c; PPD, probing pocket depth.

### 2.2 Medical Examination

Participants underwent a routine medical examination. Venous blood was obtained, and blood tests were performed, one of which included HbA1c. HbA1c levels were measured by high‐performance liquid chromatography (HLC‐723G7, Tosoh, Tokyo, Japan). Demographic data (age and sex) and information regarding current smoking status, regular dental checkups (regular visits to dentists for routine examination and cleaning), frequency of brushing, and interdental brushing were collected through a self‐administered questionnaire.

### 2.3 Dental Examination

Trained dentists examined the dental and periodontal conditions of the participants, including the number of teeth, carious status, types of prosthetic restoration, PPD, clinical attachment level (CAL), bleeding on probing (BOP), CI score, and DI score. PPD, CAL, and BOP were measured at the mesio‐buccal and mid‐buccal sites for all teeth, excluding the third molars. For the PPD and CAL measurements, the intraclass correlation coefficients were 0.78–0.90 for the three examiners and one gold‐standard examiner. The percentage of BOP was calculated from the total sites. DI and CI scores were assessed on the buccal surfaces of the upper right and left first molars, right central incisor, lower left central incisor, and the lingual surfaces of the lower right and left first molars. In case the participants were missing these teeth, DI and CI scores were assessed on the same surfaces of adjacent teeth. The highest scores were recorded as representative for each participant.

### 2.4 Quantification of Bacteria

Whole saliva samples were obtained between 9:00 and 11:00 a.m. The participants were required to abstain from eating, drinking, and brushing their teeth for 2 h before saliva collection. Masticatory stimulated saliva was collected from each participant using OMNIgene OM‐501 (DNA Genotek, Canada). Bacterial DNA was extracted from 200 µL of saliva with a QIAamp MinElute Viruses Spin (Qiagen, Venlo, the Netherlands) kit and quantified using the Mx3000P quantitative polymerase chain reaction system. The sequences of the primers for *P. gingivalis*, *A. actinomycetemcomitans*, and total bacteria have been described elsewhere (Ito et al. 2012; Nonnenmacher et al. 2004). Standard curves for each microorganism were plotted for each primer set using threshold cycle values obtained by amplification of 10‐fold serial dilutions (1–10^7^ colony‐forming units [CFU]) of previously quantified bacterial DNA. Bacterial DNA standards and sample DNA were run in duplicate, and their means were used to calculate the bacterial levels. The CFU value equivalent to the bacterial DNA of each sample was converted to a logarithm.

### 2.5 Statistical Analyses

To evaluate bivariable relationships between dental deposits and other variables, *t‐*tests and *χ* ^2^ tests were conducted to compare the two groups. We set DI = 2 and CI = 1 as reference points because these integers divide the study population most evenly. Univariable logistic regression analyses were performed to evaluate the BOP ratio, maximum PPD, maximum CAL, and the number of teeth as dependent variables. Age, sex, regular dental checkups, frequency of brushing, interdental brushing, smoking, HbA1c, BOP ratio, maximum PPD, maximum CAL, the number of teeth, maximum DI score, and maximum CI score were included among the independent variables. Based on the classification of periodontal diseases and conditions introduced in 2017 by the American Academy of Periodontology and the European Federation of Periodontology, we set BOP = 10%, PPD = 6 mm, CAL = 5 mm, and number of teeth = 20 as reference points (Tonetti et al. 2018). Multivariable logistic regression analyses were performed using a purposeful selection method to build the models for periodontal and dentitional conditions (Hosmer and Lemeshow 2000). The multivariable models included age, sex, and variables with a *p* value < 0.25 in univariable logistic regression analyses. In the final model, the adjusted odds ratios (ORs), corresponding 95% confidence interval (95% CI), and *p* values were reported. JMP v15.0.0 software (SAS Institute, Cary, North Carolina, the United States) was used for all statistical analyses, and two‐tailed *p* values < 0.05 denoted statistically significant differences.

## 3 Results

### 3.1 Bivariate Relationships Between the Variables

The periodontal conditions and the number of teeth were compared among participants classified by DI or CI scores. As shown in Table 2, BOP (%), maximum PPD, and maximum CAL of participants with DI ≥ 2 and CI ≥ 1 were significantly greater than those with DI < 2 and CI < 1, respectively. The participants with DI ≥ 2 had significantly fewer teeth than those with DI < 2, whereas the participants with CI ≥ 1 had significantly more teeth than those with CI < 1.

**Table 2:** Bivariate relationships between dependent variables and dental deposits.

	DI (max)			CI (max)		
	< 2	≥ 2		< 1	≥ 1	
Dependent variables	Mean ± SD	Mean ± SD	*p* value	Mean ± SD	Mean ± SD	*p* value
BOP (%)	9.1 ± 0.9	21.4 ± 1.1	< 0.001	9.0 ± 1.1	16.5 ± 0.9	< 0.001
Max PPD (mm)	3.7 ± 0.08	4.6 ± 0.11	< 0.001	3.5 ± 0.11	4.4 ± 0.09	< 0.001
Max CAL (mm)	5.0 ± 0.10	5.9 ± 0.14	< 0.001	4.9 ± 0.13	5.5 ± 0.11	< 0.001
Number of teeth	22.4 ± 0.3	20.8 ± 0.4	0.004	19.6 ± 0.4	23.3 ± 0.3	< 0.001

Abbreviations: BOP, bleeding on probing; CAL, clinical attachment level; CI, calculus index; DI, debris index; PPD, probing pocket depth.

The covariates were compared among participants classified by dental deposits. The participants with DI ≥ 2 were significantly older than those with DI < 2, whereas no significant difference was observed in age between the groups classified by CI (Table 3). There were no significant differences in HbA1c level as classified by dental deposits. Regarding the salivary bacterial levels, *P. gingivalis* levels in participants with DI ≥ 2 and CI ≥ 1 were significantly higher than those in participants with DI < 2 and CI < 1, respectively. *A. actinomycetemcomitans* levels in participants with DI ≥ 2 were significantly higher than those in participants with DI < 2. There were no significant differences in *A. actinomycetemcomitans* levels as classified by CI score or in total bacterial level as classified by CI or DI scores. The proportion of male participants with DI ≥ 2 and CI ≥ 1 was significantly higher than that of female participants with DI ≥ 2 and CI ≥ 1, respectively. DI score was inversely associated with regular dental checkups, frequency of brushing, and interdental brushing. CI score was inversely associated with regular dental checkups and interdental brushing but not with the frequency of brushing. There were no significant differences in current smoking status as classified by dental deposits.

**Table 3:** Bivariate relationships between the covariates and dental deposits.

	DI (max)			CI (max)		
Covariates	< 2	≥ 2	*p* value	< 1	≥ 1	*p* value
Age^a^	64.3 ± 0.6	67.1 ± 0.8	0.006	66.4 ± 0.7	64.6 ± 0.6	0.065
HbA1c^a^	5.66 ± 0.02	5.73 ± 0.03	0.064	5.69 ± 0.03	5.68 ± 0.02	0.704
Bacterial levels						
*P. gingivalis* a	2.1 ± 0.1	2.6 ± 0.1	< 0.001	2.0 ± 0.1	2.5 ± 0.1	< 0.001
*A. actinomycetemcomitans* a	0.2 ± 0.1	0.6 ± 0.1	0.046	0.3 ± 0.1	0.4 ± 0.1	0.788
Total bacteria^a^	5.8 ± 0.0	5.8 ± 0.1	0.912	5.8 ± 0.0	5.8 ± 0.0	0.352
Sex^b^						
Male	127 (53.8)	109 (46.2)	< 0.001	71 (30.1)	165 (69.9)	< 0.001
Female	304 (69.9)	131 (30.1)		198 (45.5)	237 (54.5)	
Regular dental checkups^b^						
No	293 (59.7)	198 (40.3)	< 0.001	161 (32.8)	300 (67.2)	< 0.001
Yes	138 (76.7)	42 (23.3)		108 (60.0)	72 (40.0)	
Frequency of brushing^b^						
< 2 times a day	52 (43.3)	68 (56.7)	< 0.001	40 (33.3)	80 (66.7)	0.096
≥ 2 times a day	379 (68.8)	172 (31.2)		229 (41.6)	322 (58.4)	
Interdental brushing^b^						
No	156 (51.5)	147 (48.5)	< 0.001	102 (33.7)	201 (66.3)	0.002
Yes	275 (74.7)	93 (25.3)		167 (45.4)	201 (54.6)	
Smoking^b^						
No	402 (65.2)	215 (34.9)	0.092	253 (41.0)	364 (59.0)	0.102
Yes	29 (53.7)	25 (46.3)		16 (29.6)	38 (70.4)	

Abbreviations: BOP, bleeding on probing; CAL, clinical attachment level; CI, calculus index; DI, debris index; HbA1c, hemoglobin A1c; PPD, probing pocket depth.

^a^ Data represented as mean ± SD; statistical analysis was performed by *t*‐test.

^b^ Data represented as *n* (%); statistical analysis was performed by *χ* ^2^ test.

In univariable logistic regression analyses, a higher BOP ratio was significantly associated with not receiving regular dental checkups, less frequent brushing, not using interdental brushes, smoking, deeper PPD, greater CAL, fewer teeth, and higher salivary *P. gingivalis* levels (Table 4). Deeper PPD was associated with being male, not using interdental brushes, higher BOP ratio, higher salivary *P. gingivalis* levels, and higher salivary *A. actinomycetemcomitans* levels. Greater CAL was associated with older age, male sex, less frequent brushing, not using interdental brushes, higher HbA1c level, higher BOP ratio, more teeth, higher salivary *P. gingivalis* levels, and higher salivary *A. actinomycetemcomitans* levels. Fewer teeth were associated with older age, less frequent brushing, not using interdental brushes, higher HbA1c levels, higher BOP ratios, and greater CAL.

**Table 4:** Univariable logistic regression models of periodontal and dentitional conditions.

	BOP		PPD		CAL		Number of teeth	
Parameter	OR (95% CI)	*p*	OR (95% CI)	*p*	OR (95% CI)	*p*	OR (95% CI)	*p*
Age	1.01 (0.99–1.02)	0.323	1.01 (0.99–1.03)	0.201	1.04 (1.03–1.06)	< 0.001	1.11 (1.09–1.14)	< 0.001
Sex (male)	1.35 (0.98–1.86)	0.069	1.78 (1.20–2.65)	0.004	2.03 (1.46–2.83)	< 0.001	1.01 (0.77–1.58)	0.569
Regular dental checkups (yes)	0.60 (0.42–0.87)	0.006	0.90 (0.58–1.41)	0.653	0.86 (0.61–1.22)	0.401	0.78 (0.52–1.15)	0.219
Brushing (≥ 2 times a day)	0.64 (0.43–0.95)	0.029	0.93 (0.56–1.57)	0.795	0.59 (0.39–0.89)	0.012	0.32 (0.21–0.48)	< 0.001
Interdental brushing (yes)	0.55 (0.40–0.75)	< 0.001	0.58 (0.39–0.87)	0.007	0.51 (0.38–0.70)	< 0.001	0.48 (0.34–0.68)	< 0.001
Smoking (yes)	1.81 (1.03–3.16)	0.038	1.46 (0.76–2.82)	0.258	1.67 (0.93–3.01)	0.086	1.27 (0.65–2.47)	0.486
HbA1c	1.66 (0.54–5.05)	0.374	1.02 (0.25–4.22)	0.976	1.48 (1.07–2.06)	0.019	1.59 (1.13–2.24)	0.008
BOP ( ≥ 10%)			4.39 (2.88–6.69)	< 0.001	2.63 (1.90–3.64)	< 0.001	1.52 (1.07–2.15)	0.019
PPD ( ≥ 6 mm)	4.39 (2.88–6.69)	< 0.001			ND		1.21 (0.77–1.85)	0.397
CAL ( ≥ 5 mm)	2.63 (1.90–3.64)	< 0.001	ND				2.46 (1.71–3.59)	< 0.001
Number of teeth (< 20)	1.52 (1.07–2.15)	0.019	1.21 (0.77–1.85)	0.397	2.46 (1.71–3.59)	< 0.001		
*P. gingivalis*	1.19 (1.09–1.30)	< 0.001	1.82 (1.53–2.16)	< 0.001	1.31 (1.20–1.43)	< 0.001	1.24 (0.64–2.43)	0.525
*A. actinomycetemcomitans*	1.06 (0.98–1.14)	0.146	1.15 (1.04–1.26)	0.005	1.10 (1.02–1.18)	0.010	1.03 (0.95–1.12)	0.462
Total bacteria	1.32 (0.31–5.71)	0.708	1.02 (0.77–1.34)	0.898	0.99 (0.80–1.22)	0.909	0.95 (0.75–1.21)	0.687

Abbreviations: BOP, bleeding on probing; CAL, clinical attachment level; CI, calculus index; 95% CI, 95% confidence interval; DI, debris index; HbA1c, hemoglobin A1c; ND, not determined; OR, odds ratio; PPD, probing pocket depth.

### 3.2 Multivariable Regression Models of Periodontal and Dentitional Conditions

Multivariable logistic regression analysis shows that higher BOP ratio was significantly associated with greater DI (OR = 2.51, 95% CI: 1.75–3.61), greater CI (OR = 2.18, 95% CI: 1.47–3.23), greater CAL, and fewer teeth after adjustment for potential confounding factors (Table 5). Deeper PPD was associated with greater CI (OR = 2.04, 95% CI: 1.22–3.50), higher BOP ratio, and higher salivary *P. gingivalis* levels. Greater CAL was associated with older age, being male, greater DI (OR = 1.51, 95% CI: 1.02–2.23), higher BOP ratio, fewer teeth, and higher salivary *P. gingivalis* levels. Fewer teeth were associated with older age, not receiving regular dental checkups, less frequent brushing, not using interdental brushing, greater DI (OR = 1.70, 95% CI: 1.04–2.76), smaller CI (OR = 0.13, 95% CI: 0.08–0.23), higher BOP ratio, and greater CAL.

**Table 5:** Multivariable logistic regression models of periodontal and dentitional conditions.

	BOP		PPD		CAL		Number of teeth	
Parameter	OR (95% CI)	*p*	OR (95% CI)	*p*	OR (95% CI)	*p*	OR (95% CI)	*p*
Age	0.99 (0.98–1.01)	0.270	1.00 (0.98–1.02)	0.708	1.03 (1.01–1.04)	< 0.001	1.10 (1.08–1.13)	< 0.001
Sex (male)	0.88 (0.61–1.27)	0.489	1.41 (0.90–2.20)	0.130	1.73 (1.18–2.55)	0.005	0.67 (0.41–1.08)	0.098
Regular dental checkups (yes)							0.57 (0.33–0.98)	0.043
Brushing (≥ 2 times a day)					0.83 (0.51–1.35)	0.459	0.40 (0.23–0.70)	0.001
Interdental brushing (yes)							0.51 (0.32–0.82)	0.005
Smoking (yes)	1.52 (0.80–2.88)	0.199			1.58 (0.81–3.11)	0.182		
DI (max) (≥ 2)	2.51 (1.75–3.61)	< 0.001	1.04 (0.65–1.64)	0.883	1.51 (1.02–2.23)	0.037	1.70 (1.04–2.76)	0.034
CI (max) (≥ 1)	2.18 (1.47–3.23)	< 0.001	2.04 (1.22–3.50)	0.007	1.32 (0.90–1.94)	0.156	0.13 (0.08–0.23)	< 0.001
BOP ( ≥ 10%)			3.21 (2.04–5.10)	< 0.001	1.95 (1.36–2.81)	< 0.001	1.76 (1.12–2.78)	0.015
CAL ( ≥ 5 mm)	1.94 (1.35–2.80)	< 0.001					2.20 (1.38–3.52)	0.001
Number of teeth (< 20)	1.71 (1.11–2.66)	0.015			1.94 (1.23–3.05)	0.004		
*P. gingivalis*	1.10 (1.00–1.21)	0.060	1.66 (1.41–1.99)	< 0.001	1.24 (1.12–1.36)	< 0.001		

Abbreviations: BOP, bleeding on probing; CAL, clinical attachment level; CI, calculus index; 95% CI, 95% confidence interval; DI, debris index; HbA1c, hemoglobin A1c; OR, odds ratio; PPD, probing pocket depth.

## 4 Discussion

A greater DI score was associated with a higher BOP ratio, greater CAL, and fewer teeth, whereas a greater CI score was associated with a higher BOP ratio, deeper PPD, and more teeth. Thus, although DI and CI scores were associated with more severe periodontal conditions, only the CI score was associated with a greater number of teeth.

Previous evidence suggested that increased DI scores, which reflect the amount of biofilm accumulated on the tooth surfaces, would be associated with the development of periodontitis (Lertpimonchai et al. 2017). Since bacteria in biofilm are the main cause of periodontitis (Hajishengallis 2015), an increased DI score should be associated with more severe inflammation of the periodontal tissue, including increased BOP ratio, PPD, and CAL. This is consistent with the findings that poor oral hygiene increases the risk of periodontitis by approximately 2–5 times compared to good oral hygiene (Lertpimonchai et al. 2017) and that a profound reduction in plaque, BOP ratio, and PPD was observed after strict oral hygiene (Preus et al. 2020).

Similarly to the DI score, the CI score was positively associated with a higher BOP ratio and deeper PPD. Dental calculus is known as a plaque retention factor (Roberts‐Harry and Clerehugh 2000), and the CI score was significantly correlated with the DI score in this population (*r* = 0.505). Hence, increased CI scores would lead to dental biofilm accumulation and severe inflammation in the periodontal tissue, such as increased BOP ratio and PPD. Although previous cross‐sectional studies demonstrated that deposition of calculus was associated with a decreased number of teeth (Haas et al. 2019), the CI score was associated with a greater number of teeth in the present study. Because calculus is accompanied by loss of attachment and progression to advanced disease, teeth deposited with heavy calculus have a high probability of being lost (Ramseier et al. 2017). Losing a tooth with a high CI score results in a lower CI score and fewer teeth remaining, leading to a positive association between these two indices. In fact, there were no CI 3 scores for participants with less than 15 remaining teeth in this study (data not shown). This low calculus deposition in participants with fewer remaining teeth may explain the discrepancy between our findings and previous evidence (Haas et al. 2019). It is also worth noting that there are differences between the sites of debris and calculus deposition. Calculus is frequently formed at sites close to the major salivary gland orifices, that is, mandibular incisors and maxillary molars (Dawes 2006), whereas supragingival biofilm can develop on any teeth. Loss of teeth at frequent calculus deposition locations may reduce the number of remaining teeth and lower the CI score but not the DI score, which explains the different relationship of DI and CI to the number of teeth. However, future studies are necessary to reveal the exact reason for the relationship between these indices.

This study has several limitations. The average age of the participants (65.0 ± 12.0 years) in the Goto Islands was relatively high, and advanced periodontitis was likely to be the main cause of tooth loss. If the participants had been younger, the reasons for the participant's tooth loss and oral hygiene status would have been different. Therefore, the findings for this population may be different from those that would result for younger populations in urban areas, yet may suggest an emerging trend in Japan's super‐aging society. Information regarding antibiotic treatment was not collected. This may affect salivary *P. gingivalis* and *A. actinomycetemcomitans* levels. A history of dental or periodontal treatment was not collected in this study. The treatment status at the time of the survey may have affected the results of the periodontal tissue examination. The present cross‐sectional study was unable to establish a causal relationship between oral hygiene status and periodontal and dentitional conditions. The impact of dental calculus on periodontal and dentitional conditions would be revealed by a longitudinal study of the same cohort.

Because debris and calculus deposition were inversely related to the number of teeth, either should be more closely related to tooth loss in the longitudinal study. Identifying the key risk factors for tooth loss is important in developing strategies for better oral health management in a super‐aging society.

## Author Contributions

M.O. contributed to the study conceptualization, data curation, formal analysis, investigation, and writing of the manuscript (original draft). M.K. contributed to the data curation, formal analysis, and investigation. K.H. and M.L.F.C. contributed to the methodology and investigation. S.I., Y.Y., Y.O., S.S., and E.S. contributed to the data curation and methodology. T.M., A.K., and T.S. contributed to the project administration, supervision, and writing of the manuscript (review and editing). A.Y. contributed to the study conceptualization, methodology, project administration, supervision, and writing of the manuscript (review and editing). All authors read and approved the final version of the manuscript.

## Ethics Statement

This study was conducted in accordance with the tenets of the Declaration of Helsinki 2013, and the protocol was approved by the Nagasaki University Ethics Committee (#14051404‐16). All participants received verbal and written information before their participation and provided written informed consent.

## Conflicts of Interest

The authors declare no conflicts of interest.

## Data Availability

The data that support the findings of this study are available on request from the corresponding author.
